# Knowledge among patients with Hepatitis C initiating on direct-acting antiviral treatment in rural Rwanda: A prospective cohort study

**DOI:** 10.1080/16549716.2021.1953250

**Published:** 2021-08-04

**Authors:** Dale A Barnhart, Innocent Kamali, Francoise Nyirahabihirwe, Carol Mugabo, Jean de la Paix Gakuru, Mariam Uwase, Esdras Nizeyumuremyi, Tumusime Musafiri, Jean de Dieu Gatete, Jean Damascene Makuza, Fredrick Kateera, Bethany Hedt-Gauthier, Jean d’Amour Ndahimana

**Affiliations:** a Department of Global Health and Social Medicine, Harvard Medical School, Boston, MA, USA; b Partners In Health-Rwanda/Inshuti Mu Buzima, Rwinkwavu, Rwanda; c School of Population and Public Health, University of British Columbia, Vancouver, BC, Canada; d STIs and OBBI Division, Rwanda Biomedical Center, HIV/AIDS, Kigali, Rwanda; e Department of Biostatistics, Harvard T.H. Chan School of Public Health, Boston, MA, USA

**Keywords:** Hepatitis C, Rwanda, health knowledge, attitudes, practice, direct acting antivirals, treatment initiation

## Abstract

**Background:**

Curative direct-acting antiviral treatment (DAA) has made it plausible to implement hepatitis C elimination interventions. However, poor hepatitis C knowledge among patients could impede the effectiveness of screening and treatment programs.

**Objective:**

We assessed knowledge on hepatitis C among rural Rwandans initiating DAA treatment for hepatitis C in a prospective cohort.

**Methods:**

We administered 15 true-false statements before treatment initiation and during one follow-up visit occurring either 1 or 2 months after treatment initiation. We assessed the average number of correct responses per patient, the proportion of correct responses to individual statements, pre-treatment predictors of knowledge, and whether post-initiation knowledge was associated with time since treatment initiation, quality of care, or adherence.

**Results:**

Among 333 patients who answered knowledge questions before treatment initiation, 325 (97.6%) were re-assessed at a post-initiation visit. Pre-initiation, 72.1% knew hepatitis C was curable, 61.9% knew that hepatitis C could cause liver damage or cancer, and 42.3% knew that people with hepatitis C could look and feel fine. The average number of correct responses was 8.1 out of 15 (95% CI: 7.8–8.5), but was significantly lower among those with low educational attainment or with low literacy. Post-initiation, correct responses increased by an average of 2.0 statements (95% CI: 1.6, 2.4, *p*-value <0.001). Many patients still mistakenly believed that hepatitis C could be transmitted through kissing (66.5%), eating utensils (44.1%), handshakes (34.8%), and hugs (34.8%). Post-initiation knowledge is inversely associated with self-reported quality of care and unassociated with self-reported adherence.

**Conclusion:**

Although knowledge improved over time, key gaps persisted among patients. Accessible public education campaigns targeted to low-literacy populations emphasizing that hepatitis C can be asymptomatic, has severe consequences, and is curable could promote participation in mass screening campaigns and linkage to care. Visual tools could facilitate clinician-provided patient education.

## Background

Chronic hepatitis C affects 71 million people globally with 400,000 people dying of hepatitis C-related causes annually [1]. The discovery of easy-to-deliver oral direct-acting antiviral (DAA) treatments with cure rates of over 90% of chronic hepatitis C infections [2] has made hepatitis C elimination plausible and led the World Health Organization (WHO) to call for a 65% reduction in global mortality from hepatitis C by 2030 [3]. Although costs of testing and treatment remain major barriers to optimal coverage, particularly in low-and-middle-income settings, by 2017 over 5 million patients globally had been initiated to DAA treatment, and access to DAAs is expected to increase over time [4–6].

As access to DDAs increases, patients’ knowledge about hepatitis C could influence uptake of hepatitis screening, linkage to treatment, and treatment outcomes, thereby impacting the likelihood of successful elimination campaigns. Participant’s disease-related knowledge has been associated with increased participation in screening campaigns for many diseases, including cervical cancer [7], hepatitis B [8], and HIV [9]. Among patients enrolled in hepatitis C treatment programs, higher patient knowledge has been associated with reduced loss to follow-up [10], while other educational interventions, such as single-session group educational interventions and multi-session one-on-one educational interventions, have been associated with increased willingness to initiate treatment, reduced time to treatment initiation, increased adherence, and increased likelihood of sustained virologic response (SVR) [11–13].

However, patients’ knowledge of hepatitis varies by context. In the USA, over 90% of patients diagnosed with hepatitis C knew that people infected with hepatitis C can look and feel fine compared to less than 50% of patients in Egypt [14,15]. Similarly, in China, over 90% of patients in urban Beijing know that hepatitis C can cause cirrhosis compared to less than 50% in rural Hebei [16]. Relatively little research on hepatitis C knowledge has been conducted in Africa, and most has been confined to Egypt [15] or conducted among health-care workers rather than patients [17]. In Rwanda, where 6.8% of participants of mass screening programs screened positive of hepatitis C antibodies [18], the national government has established a plan to eliminate hepatitis C by 2024 through wide-spread screening programs and free access to DAA treatment for all Rwandans [19]. Although qualitative interviews suggest that lack of knowledge among patients served as a barrier to successful hepatitis C treatment [20], no previous studies have assessed the level of hepatitis C knowledge among Rwandan patients. We assessed hepatitis C knowledge among Rwandans with chronic hepatitis C initiated on DAA treatment at rural health centers and assessed changes in knowledge after their initial clinical consultation and treatment initiation.

## Methods

### Study setting

Patients in this prospective cohort were enrolled from government-led health facilities in two rural Rwandan districts, Kayonza and Kirehe, supported by an international NGO called Partners In Health/Inshuti Mu Buzima (PIH/IMB) that has been supporting health system strengthening in Rwanda since 2005. In 2019 and 2020, PIH/IMB supported government-operated mass screening campaigns in Kirehe and Kayonza. These screening campaigns were open to all Rwandans aged 15 years and above. In particular, PIH/IMB in partnership with Ministry of Health facility staff, supported linkage to care for patients with chronic hepatitis C who were eligible for DAA treatment, defined as having a detectable viral load (hepatitis C RNA ≥15 IU/mL) by implementing a novel mobile hepatitis clinic as described in detail elsewhere [21]. Briefly, this mobile clinic approach was designed to facilitate access to hepatitis treatment by decentralizing hepatitis care to primary-level health centers. Up to 10 hepatitis C patients were invited to their closest health center on a given mobile clinic day and offered same-day pre-initiation laboratory exams, patient education, clinical consultation, and DAA treatment initiation at their nearest health centers. Although this delivery approach was novel, the care provided to patients followed Rwanda’s national hepatitis guidelines and included clinician-provided patient education. Information was provided to patients during individual consultation as well as through a group education session prior to the treatment dispensing. During these sessions, clinicians discussed themes including hepatitis C transmission and prevention, length of hepatitis C treatment and potential side effects, the importance of treatment adherence, the importance of SVR testing to verify a cure, and risk factors that can accelerate progression of liver damage. However, there was no standard curriculum for either individual counseling or group education sessions.

### Study population

We enrolled patients aged ≥18 years diagnosed with chronic hepatitis C and initiating DAA treatment for the first time at 16 primary-level health centers. Our participants were diagnosed with hepatitis C through previous national screening campaigns and were identified from patients attending a hepatitis mobile clinic campaign occurring from July 2020 to September 2020. During this time, within-district COVID-19-related travel restrictions had been lifted, which meant that neither travel from the communities to the local health center hosting the mobile clinic services nor provision of routine clinical care were impacted by COVID-19 related travel restrictions. Our target sample size was 330 patients.

### Data collection

Patients were recruited at health facilities during mobile hepatitis clinic days. After arrival at the health center but before patient education or consultation with the clinician, patients were approached by study-trained staff to confirm patient eligibility, administer an informed consent process, and upon enrollment, administer a baseline questionnaire that assessed socio-demographics, knowledge of hepatitis C, and other topics. All questionnaires were administered verbally in Kinyarwanda, and data was gathered using the REDCap mobile app [22]. When patients returned to the health facility to pick up their DAA medication for their second and third month of treatment, study staff administered two other follow-up interviews at approximately 30 and 60 days after treatment initiation. Questions on hepatitis knowledge were re-administered at the 30-day follow-up, with patients who did not complete their 30-day follow-up interview responding to knowledge questions at their 60-day follow-up. Due to inter-district travel restrictions related to the COVID-19 pandemic, the 30-day follow-up visit was canceled for patients in Kayonza district such that all patients from Kayonza completed their second set of knowledge questions at the 60-day interview.

### Hepatitis knowledge

To assess hepatitis knowledge, we first identified relevant true/false statements from the literature [14,15,17]. Because injecting drug use is a smaller driver of blood-born infections in Africa than elsewhere [23], we excluded statements from this literature related to injecting drug use, leaving us with 15 items. To assess patient knowledge about DAA treatment, which was not widely available at the time of these previous studies, we replaced the statement ‘If someone is infected with hepatitis C, they will most likely carry the virus all their lives,’ used by Dennison et al. and Sultan et al. with the statement ‘There is a treatment to cure hepatitis C.’ For all statements, response options included, ‘True’, ‘False’, and ‘Don’t know’. These statements were translated from English to Kinyarwanda by two native Kinyarwanda speakers, and the two translations were compared and harmonized by a third native Kinyarwanda speaker. Back-translations into English were conducted by two additional native Kinyarwanda speakers who are fluent in English, and the full translation team met to discuss the back-translated versions and resolve any discrepancies. The full set of 15 questions used and their Kinyarwanda translations can be found in Appendix A.

### Statistical analysis

Patient-related variables were summarized using frequencies for categorical variables and medians and interquartile ranges for continuous variables. Socioeconomic status was assessed by Ubudehe, which is a categorization used by the Rwandan government to assess eligibility for public support, with category 1 used for households in acute poverty and category 3 reflecting households with a stable livelihood and source of income [24].

Pre-treatment initiation hepatitis knowledge scores was defined as the total number of correct responses out of 15 questions administered prior to the treatment initiation visit. Responses of ‘don’t know’ were coded as incorrect. Because missingness for individual knowledge items was minimal (<1% for all 15 statements), we also coded missing responses as incorrect when calculating the summary score. We reported average knowledge scores and 95% confidence intervals for the overall patient population and for subpopulations defined by socioeconomic characteristics, sources of hepatitis knowledge, and loss to follow-up status, where loss to follow-up was defined as missing both the 30-day and 60-day follow-up surveys. To assess whether knowledge level differed by these characteristics, we modeled the number of correct responses (maximum of 15) as a continuous outcome and used a likelihood ratio test comparing a null linear regression model to a model that included the characteristic of interest. We also calculated the percentage of patients who correctly responded to each of the 15 individual items used to assess hepatitis C knowledge, excluding individuals with missing data for individual items.

Among patients who completed knowledge questions at baseline and at least one of the follow-up visits, we calculated the mean number of correct responses pre- and post-treatment initiation and tested for overall change using a paired t-test. We assessed whether either post-treatment initiation knowledge or change in knowledge from pre-treatment initiation to post-treatment initiation was associated with timing of the follow-up visit, quality of care, and DAA adherence using linear regression. Associations were assessed using both an unadjusted linear regression model and an adjusted model that included sex, age, and education. Quality of care was assessed using the patients’ average response to the 14-item Kinyarwanda-Communication Assessment Tool (K-CAT) and using patient’s overall assessment of the care provided ranked using a 5-point Likert scale [25]. Adherence was assessed using the Single-Item Self-Report tool, a 6-point Likert scale with options ranging from ‘very poor’ to ‘excellent’ adherence [26].

To assess improvement in knowledge of individual items, we plotted the percentage of patients with a correct response for each item at treatment initiation and at the post-treatment initiation follow-up and reported the percentage point increase in correct responses of each statement. For each item, we limited the sample to individuals who had responded to that item at both the treatment initiation visit and at the post-treatment initiation follow-up visit.

### Ethics

All patients provided an informed written consent. This study was approved by the Inshuti Mu Buzima Research Committee and by the Rwanda National Ethics Committee (IRB 00001497) and conducted in accordance with national regulations.

## Results

A total of 340 patients were enrolled. However, due to a data upload error, data for only 333 patients with baseline questionnaire data were analyzed. The majority (85.0%) of study participants were enrolled from Kirehe district; about two-thirds (64%) were female, and the median age among the participants was 63 years (IQR: 49–73 years). Only one in four of the study participants had completed primary school, but over half (55%) reported being able to read and write. Prior to treatment initiation, participants reported that the most common sources of information about hepatitis C were provided by facility-based health care workers (46%), with radio, family and friends, and community meetings being less common sources of information.

Missing data for hepatitis knowledge items was rare, with 327 patients (98.2%) answering all knowledge questions and no patient missing data on more than 3 knowledge items. At baseline, the average number of correct responses out of 15 questions was 8.1 (95% CI: 7.8–8.5). On average, patients coming from Kirehe answered more questions correctly than those coming from Rwinkwavu (8.5 vs. 6.1, *p* < 0.001). Men also answered more questions correctly compared to women (8.6 vs. 7.8, *p* = 0.030), and those who had completed primary school or could read answered more questions correctly than their peers (Table 1). There were no significant differences in pre-treatment knowledge by socioeconomic status or source of knowledge.

**Table 1.** Demographic characteristics of study population (*N* = 333)

	*N*	%
District of residence		
Rwinkwavu	50	(15.0%)
Kirehe	283	(85.0%)
Sex		
Female	213	(64.0%)
Male	120	(36.0%)
Age, categorized		
<45	69	(20.7%)
45–59	64	(19.2%)
60–74	137	(41.1%)
>75	63	(18.9%)
Education^a^		
No schooling	117	(35.2%)
Primary level, incomplete	131	(39.5%)
Primary level, complete	59	(17.8%)
Some secondary school or higher	25	(7.5%)
Literacy		
Reads and writes	179	(53.8%)
Reads only	16	(4.8%)
Not literate	138	(41.4%)
Ubudehe		
Category 1	72	(21.6%)
Category 2	129	(38.7%)
Category 3	130	(39.0%)
Uncategorized	2	(0.6%)
Sources of hepatitis knowledge^b^		
Facility-based health worker	153	(45.9%)
Radio	36	(10.8%)
Family & friends	32	(9.6%)
Community meeting	26	(7.8%)
Home visit by health worker	11	(3.3%)
Print source	3	(0.9%)
Television	2	(0.6%)

^a^Education level not reported for one respondent. ^b^Participants could identify multiple sources of knowledge.

**Figure 1.**
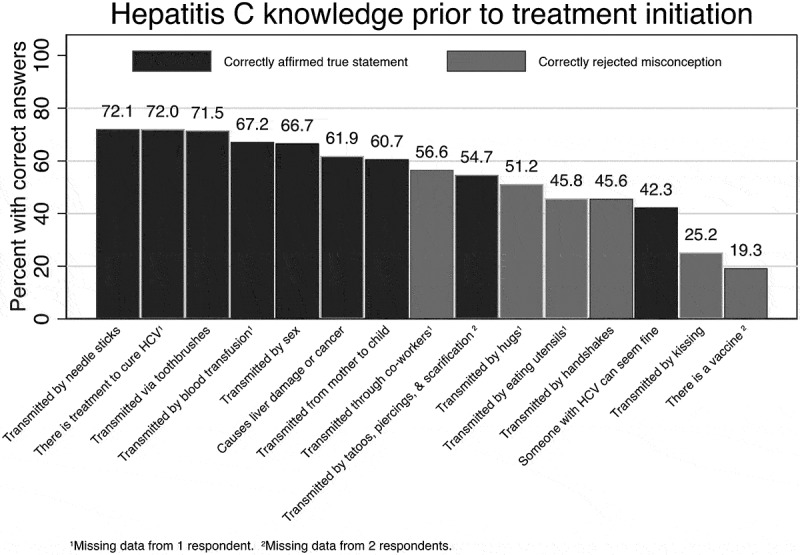
Hepatitis C knowledge prior to treatment initiation (*N* = 333)

Prior to treatment initiation, patients were most likely to know that hepatitis C can be transmitted by needle sticks (72.1%), that there is a treatment to cure hepatitis C (72.0%), and that hepatitis C can be transmitted by sharing toothbrushes (71.5%) (Figure 1). Fewer patients rejected the misconceptions that hepatitis C could be transmitted by kissing (25.2%) and that there is a vaccine for hepatitis C (19.3%). In general, patients were more likely to correctly affirm to true statements about hepatitis C than they were to reject false statements.

Knowledge questions were re-administered to 325 patients (97.6%) during at least one follow-up visit. There were no differences in baseline knowledge scores among those who returned for a second interview and compared to those who did not (Table 2). Due to COVID-related disruptions to data collection in Kayonza, all of the 277 patients whose knowledge was re-assessed during the first follow-up visit were from Kirehe district while 47 of the 48 patients whose knowledge was re-assessed during the second follow-up visit were from Kayonza. Among these 325 patients, the average number of correctly answered questions increased from 8.1 prior to treatment initiation to 10.1 (95% CI: 9.8, 10.4) after treatment initiation, with an average change of 2.0 (95% CI: 1.6, 2.4, *p*-value <0.0001).

**Table 2.** Average number of correct responses to 15 questions about hepatitis C (*N* = 333)

	Score	95% CI	*p*-value
District of residence			<0.001
Rwinkwavu	6.1	5.2,6.9	
Kirehe	8.5	8.1,8.8	
Sex			0.030
Female	7.8	7.4,8.3	
Male	8.6	8.0,9.2	
Age			<0.001
<45	9.4	8.6,10.1	
45–59	8.9	8.2,9.7	
60–74	7.9	7.3,8.4	
>75	6.5	5.7,7.2	
Education^a^			<0.001
No schooling	7.1	6.5,7.6	
Primary level, incomplete	8.5	7.9,9.0	
Primary level, complete	9.1	8.3,9.9	
Some secondary school or higher	8.8	7.6,10.1	
Literacy			0.005
Reads and writes	8.6	8.2,9.1	
Reads only	7.4	5.9,9.0	
Not literate	7.5	7.0,8.0	
Ubudehe^b^			0.113
Category 1	7.5	6.7,8.2	
Category 2	8.4	7.9,9.0	
Category 3	8.2	7.6,8.7	
**Sources of hepatitis knowledge**			
Facility-based health worker			0.268
No	7.9	7.5,8.4	
Yes	8.3	7.8,8.8	
Radio			0.416
No	8.1	7.7,8.4	
Yes	8.5	7.5,9.6	
Family & friends			0.673
No	8.1	7.7,8.5	
Yes	8.3	7.2,9.5	
Community meeting			0.185
No	8.2	7.8,8.5	
Yes	7.3	6.1,8.6	
Home visit by health worker			0.434
No	8.1	7.8,8.5	
Yes	7.4	5.4,9.3	
Print source			0.634
No	8.1	7.8,8.5	
Yes	9.0	5.3,12.7	
TV			0.866
No	8.1	7.8,8.5	
Yes	8.5	4.0,13.0	
Lost to follow-up			0.904
No	8.1	7.8,8.5	
Yes	8.2	6.0,10.5	

^a^Education missing for one individual, *N* = 332. ^b^Individuals without an ubudehe category were excluded from analysis, *N* = 331.

Compared to patients whose knowledge was reassessed during their first follow-up visit, patients whose knowledge was re-assessed during the second follow-up visit reported 1.0 more correct responses (95% CI: 0.2, 1.7, *p* < 0.001) and experienced greater improvements from their pre-treatment initiation knowledge (3.4, 95% CI: 2.3–4.5) after adjusting for differences in sex, age, and education level (Table 3). Higher patient-reported quality of care, measured both with the K-CAT and the patient’s overall assessment of care, was significantly associated with worse post-treatment knowledge in both the crude and adjusted models but was not associated with change in pre- to post-treatment initiation knowledge level. Generally, self-reported treatment adherence was high with only 38 (11.7%) reporting very poor, poor, or fair adherence, 68 (20.9%) reporting good adherence, 136 (41.9%) reporting very good adherence, and 83 (25.5%) reporting excellent adherence. However, neither post-treatment initiation knowledge nor change in knowledge were significantly associated with treatment adherence in the adjusted models, although post-treatment initiation knowledge was significantly associated with having good or very-good adherence compared to very poor, poor, or fair in the unadjusted model.

**Table 3.** Associations between post-treatment initiation hepatitis c knowledge and timing of assessment, quality of care, and patient adherence (*N* = 325)

	*N* (%)/Mean (SD)	**Post-treatment initiation hepatitis C knowledge**						**Change in hepatitis C knowledge since treatment initiation**					
		Crude *N* = 325			Adjusted *N* = 324^a^			Crude *N* = 325			Adjusted *N* = 324^a^		
		β	95% CI	*p*-value	β	95% CI	*p*-value	β	95% CI	*p*-value	β	95% CI	*p*-value
Time of follow-up visit				0.015			0.010			<0.001			<0.001
First follow-up visit	277 (85.2%)	ref.			ref.			ref.			ref.		
Second follow-up visit	48 (14.8%)	1.0	[0.2,1.8]		1.0	[0.2,1.7]		3.5	[2.5,4.6]		3.4	[2.3,4.5]	
K-CAT Score, range 1–5	4.0 (0.8)	−0.6	[−0.9,-0.2]	0.002	−0.6	[−0.9,-0.3]	0.003	−0.2	[−0.7,0.3]	0.433	−0.2	[−0.7,0.3]	0.325
Overall satisfaction				<0.001			<0.001			0.175			0.154
Poor, fair, or good	93 (28.6%)	ref.			ref.			ref.			ref.		
Very good	115 (35.4%)	0.2	[−0.5,0.9]		0.4	[−0.2,1.1]		0.5	[−0.5,1.5]		0.4	[−0.6,1.4]	
Excellent	117 (36.0%)	−1.3	[−2.0,-0.6]		−1.2	[−1.9,-0.6]		−0.3	[−1.3,0.6]		−0.5	[−1.4,0.5]	
Self-reported Adherence				0.045			0.086			0.196			0.238
Very poor, poor, or fair	38 (11.7%)	ref.			ref.			ref.			ref.		
Good	68 (20.9%)	1.1	[0.1,2.1]		1.0	[−0.0,1.9]		0.9	[−0.5,2.3]		0.9	[−0.5,2.3]	
Very good	136 (41.9%)	1.1	[0.2,2.1]		0.7	[−0.2,1.6]		0.2	[−1.1,1.4]		0.1	[−1.2,1.3]	
Excellent	83 (25.5%)	0.4	[−0.6,1.4]		0.2	[−0.8,1.1]		−0.3	[−1.6,1.1]		−0.2	[−1.6,1.1]	

^a^Models are adjusted for sex, age, and education. Adjusted models are missing one individual with no data on education.

**Figure 2.**
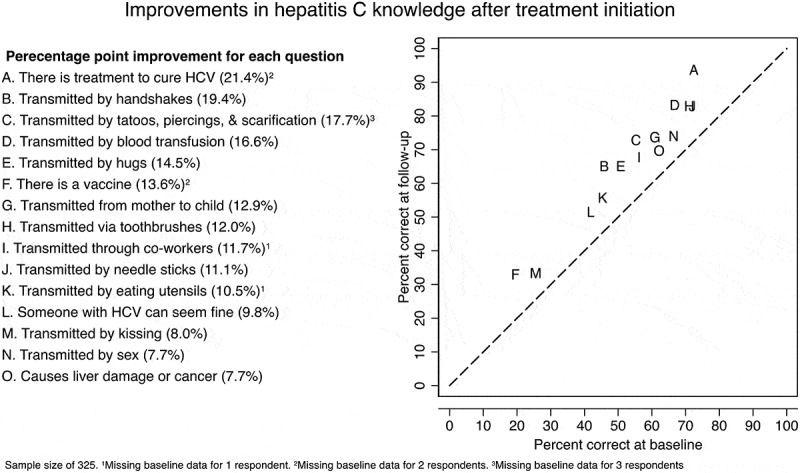
Improvements in hepatitis C knowledge after treatment initiation (*N*= 325)

When we investigated changes in correct responses to individual items in our knowledge assessment scale, we observed that patients’ knowledge improved across all items (Figure 2). Knowing that there is a treatment to cure hepatitis C was both the single item that most patients were likely to answer correctly (94.5%) and the item with the greatest improvement from pre-treatment initiation (21.4%). Patients were also improved in recognizing blood transfusion, tattoos, piercings, and scarifications as risks for hepatitis C transmission and in rejecting casual contacts like hugs and handshakes as avenues for transmission. Even after treatment initiation, many patients still mistakenly believed that hepatitis C could be transmitted through casual contacts like kissing (66.5%), shared eating utensils (44.1%), handshakes (34.8%), and hugs (34.8%). Fewer than 60% of the patients knew that there is no vaccine for hepatitis C, and that someone with hepatitis C can look and feel fine.

## Discussion

Overall, knowledge about hepatitis C was higher than expected among patients initiating DAA treatment for hepatitis C in rural Rwanda, but still suboptimal. Prior to treatment initiation, correct responses among our patients were consistently higher than has been previously reported among health care workers in Malawi [17], but lower than what has previously been reported among individuals living with hepatitis C in Egypt and the USA for all statements expect awareness of sexual transmission and transmission from mother to child [14,15] (see Appendix A for comparison of correct responses across studies). However, on average patients correctly responded to only 54% of statements (8.1 out of 15) prior to treatment initiation, supporting previous qualitative research that had identified low levels of knowledge among patients as a barrier to successful hepatitis C treatment in Rwanda [20]. We observed a significant difference in baseline knowledge levels comparing men and women. Although educational attainment for Rwandan men and women under the age of 25 is very similar, among Rwandans over 45, women are over 1.5 times more likely to have never received formal education [27]. Because increased age is a strong risk factor for hepatitis C in Rwanda, our study population was largely composed of older individuals who would have been most affected by this educational disparity. We believe that the predominance of women in our study primarily reflects a structural gender imbalance in the Rwandan population, which is pronounced in older age groups [27], because previous research in this setting has demonstrated similar linkage to care rates among men and women [21].

Even after treatment initiation, several common misconceptions about hepatitis C persisted. First, most patients believed that a vaccine existed for hepatitis C, which we believe could reflect both confusion between hepatitis B and hepatitis C and confusion between preventative vaccines and treatments. At both baseline and treatment initiation, many patients believed that causal contact, like kissing, hugs, and handshakes, could transmit hepatitis C. Combatting these misconceptions could be important for preventing the development of hepatitis C–related stigma.

Because our patients were referred for hepatitis treatment from voluntary mass screening campaigns that should have provided hepatitis education, we would expect them to have higher hepatitis knowledge than the general Rwandan population. However, we were surprised that even among patients presenting for DAA treatment initiation, only 72.1% were aware that hepatitis C was curable, 61.9% know that hepatitis C could cause liver damage or cancer, and less than half knew that patients with hepatitis C could look and feel fine. Because community-based educational campaigns have previously been linked to successful mass hepatitis screening campaigns [28], the Rwandan Ministry of Health may wish to use additional educational outreach that emphasizes the asymptomatic nature of hepatitis C, the long-term consequences, and the availability of effective treatment as this knowledge could motivate individuals to participating in screening. Because we found that lower educational attainment and limited literacy were associated with less hepatitis C knowledge, any educational campaigns should be accessible to populations with low literacy. In our sample, only 10.8% of the patients reported learning about hepatitis C through the radio, 7.8% through community meetings, and only 3.3% through home visits by health workers, including community health workers. These underutilized strategies could be effective strategy for reaching out to low-literacy populations prior to future mass screening campaigns.

After treatment initiation, we did observe patients reporting on average an additional 2 questions correct, or a 13 percentage-point increase in knowledge. The magnitude of this improvement is small, but comparable to what has been observed in previous educational interventions [29–31]. Although our clinical team placed a relatively high emphasis on patient education, the process was not standardized and there was limited time to dedicate to patient education while also providing testing and clinical services to the patients. The development of a job aid, such as an illustrated flip book, could help standardize the information provided to patients during treatment initiation, facilitate group education sessions, and still be accessible to patients with limited literacy. As Rwanda is currently task shifting hepatitis treatment initiation from district hospitals to primary-level health centers, this tool could also be used to empower nurses to provide correct and complete information for the patients. Encouragingly, we did observe that post-treatment knowledge was higher among those evaluated at their second follow-up visit than among those evaluated at their first. Although the interpretation of this finding is complicated by the collinearity between timing of the re-assessment and district, it does suggest that gains in hepatitis C knowledge were not lost over time and may suggest continued improvements in knowledge over the course of treatment. Surprisingly, we found that neither patient-provider communication nor patient-rated quality of care were associated with improvements in hepatitis C knowledge and that they were inversely associated with hepatitis C knowledge during the post-initiation visit. During the initial validation of the Kinyarwanda Communication Assessment Tool, it was observed that cultural factors could complicate the interpretation could complicate the interpretation the tool if patients perceived clinicians as authority figures and were therefore reluctant to provide negative feedback. However, this inverse association could also reflect that quality of care was rated very highly overall and we may not have observed enough variation in satisfaction to observe the expected association. Because the novel mobile clinic approach used by our team is associated with substantial time and cost savings for patients [21], high self-reported satisfaction could also primarily reflect high patient satisfaction with the overall approach used by our team rather than specifically reflecting patient-provider communication.

Our study had a few limitations. First, our knowledge assessment was conducted among rural Rwandan who had already screened positive for hepatitis C and linkage to care. Due to these patients’ elevated engagement and interactions with hepatitis C care, we hypothesize that their knowledge levels would be higher than other rural Rwandans of a similar age. Our findings may not be generalizable to younger or more urban populations, who would typically have higher access to both education and information sources. Second, although our knowledge scale was constructed using items identified from the literature and underwent careful translation and back-translation, it has not been previously used in Rwanda or validated. When asking about the existence of a medical cure for hepatitis C in Rwanda or similar settings, future studies should explicitly distinguish between a scientific medical treatment as opposed to traditional cures, which are common. Additionally, unlike previous studies, we did not observe associations between knowledge and either loss to follow up or self-reported treatment adherence. However, this finding could point to low power to detect an effect due to the relatively low levels of loss to follow up (2.5%) and poor treatment adherence in our study (11.7%) rather than suggest that patient knowledge is not important. Finally, our analysis did suffer some from some minimal missing data, including both item-level missingness in the knowledge scale items and the loss of seven baseline surveys.

### Conclusions

This study evaluated hepatitis C knowledge among Rwandans with chronic hepatitis C before and after initiation of direct-acting antiviral treatment at rural health centers. We identified gaps in patient knowledge that could affect hepatitis C screening uptake among the general population and linkage to care among patients, including limited awareness hepatitis C can be asymptomatic, has severe consequences, and is curable. Public education campaigns that are accessible to low-literacy populations and strategic patient education among those who present for hepatitis C treatment should be considered to enhance case detection, linkage to care, and adherence to treatment.

## Paper context

This prospective cohort is the first study to quantitatively assess Rwandans’ hepatitis C knowledge. Pre-treatment knowledge gaps among rural Rwandans initiating treatment for hepatitis C highlight the need for mass education campaigns that are accessible to low-literacy populations and emphasize the asymptomatic nature, severe consequences, and curability of hepatitis C. Persistent gaps in knowledge after treatment initiation suggest that clinicians may need job aids to support patient education.

## Appendix A. Hepatitis C Knowledge tool. Response options were True (Nibyo), False (Sibyo), and Don’t know (Sinzi)

**Table:**

**Item, English**	**Item, Kinyarwanda**	**Source & Adaptions**	**Percent of respondents with correct response**			
			Baseline among Rwandan patients starting hepatitis C treatment (Barnhart 2020)	Baseline levels among health care workers in Malawi (Mtengezo 2016)	Baseline levels among hepatitis C patients in Egypt (Sultan 2018)	Baseline levels in among individuals with hepatitis C in USA (Denniston 2012)
Infection with hepatitis C can cause liver damage or cancer.	Kwandura indwara y’umwijima wo mu bwoko bwa C bishobora gutuma umwijima wangirika cyangwa bigatuma umuntu arwara kanseri.	Sultan 2018; Adapted from Denniston 2012 ‘Infection with hepatitis C can cause the liver to stop working.’	61.9	NA	72.9	NA
Someone with hepatitis C can look and feel fine.	Umuntu urwaye indwara y’umwijima yo mu bwoko bwa C ashobora kugaragara nk’umuntu ufite ubuzima bwiza kandi akumva amerewe neza.	Sultan 2018; Denniston 2012	42.3	NA	47.7	90.2
Hepatitis C can be transmitted by getting a blood transfusion from an infected donor.	Indwara y’umwijima yo mu bwoko bwa C ushobora kuyandura uramutse uhawe amaraso yatanzwe n’umuntu uyirwaye.	Sultan 2018, Mtengezo 2016; Denniston 2012	67.2	65.8	86.4	90.8
Hepatitis C can be transmitted by shaking hands with someone who has hepatitis C	Umuntu ashobora kwandura indwara y’umwijima yo mu bwoko bwa C mu gihe ahanye ibiganza n’ umuntu wanduye agakoko gatera umwijima wo mu bwoko bwa C.	Sultan 2018; Denniston 2012	45.6	NA	76.6	84.6
Hepatitis C can be transmitted by kissing someone who has hepatitis C	Umuntu ashobora kwandura indwara y’umwijima yo mu bwoko bwa C mu gihe asomanye n’umuntu wayanduye	Sultan 2018; Denniston 2012	25.2	18.7	76.9	68.1
Hepatitis C can be transmitted by having sex with someone who has hepatitis C	Umuntu ashobora kwandura indwara y’umwijima yo mu bwoko bwa C mu gihe akoranye imibonano mpuzabitsina n’umuntu wayanduye	Sultan 2018, Mtengezo 2016; Denniston 2012	66.7	47.9	19.5	63.8
Hepatitis C can be transmitted by being born to a woman who had hepatitis C when she gave birth	Umubyeyi wanduye indwara y’umwijima yo mu bwoko bwa C ashobora kuyanduza umwana mu gihe cyo kumubyara.	Sultan 2018; Denniston 2012; Mtengezo 2016;	60.7	42.0	31.3	57.1
Hepatitis C can be transmitted by using an infected person’s toothbrush	Umuntu ashobora kwandura indwara y’umwijima yo mu bwoko bwa C mu gihe akoresheje uburoso bw’amenyo bw’undi muntu wayanduye	Sultan 2018	71.5	NA	83.0	NA
Hepatitis C can be transmitted by being stuck with a needle or sharp instrument that has hepatitis C-infected blood on it such as razors, blades, etc.	Umuntu ashobora kwandura indwara y’umwijima yo mu bwoko bwa C mu gihe akomerekejwe n’urushinge, urwembe nibindi bikoresho bityaye biriho amaraso arimo agakoko gatera umwijima wo mubwoko bwa C.	Sultan 2018 – adapted to remove reference to cupping (Hijama). Denniston 2012.	72.1	NA	87.5	95.7
Hepatitis C can be transmitted by working with someone who has hepatitis C	Umuntu ashobora kwandura indwara y’umwijima yo mu bwoko bwa C mu gihe ari gukorana n’umuntu wayanduye.	Sultan 2018, Denniston 2012	56.6	NA	65.0	74.9
Hepatitis C can be transmitted by hugging a person with hepatitis C	Umuntu ashobora kwandura indwara y’umwijima yo mu bwoko bwa C mu gihe ahoberanye nuwayanduye	Mtengezo 2016	51.2	9.8	NA	NA
Hepatitis C can be transmitted by sharing plates, cups, and utensils	Umuntu ashobora kwandura indwara y’umwijima yo mu bwoko bwa C mu gihe akoresheje isahani, igikombe cyangwa ibindi bikoresho byakoreshejwe nuwayanduye	Mtengezo 2016	45.8	13.5	NA	NA
Hepatitis C can be transmitted by tattoos, ear piercings, or scarification	Umuntu ashobora kwandura indwara y’umwijima yo mu bwoko bwa C mu gihe yiyandikishijeho amagambo cyangwa ibishushanyo ku mu biri we, mu gihe yitoboje amatwi cyangwa bamuciye indasago.	Mtengezo 2016. Adapted to include traditional scarification.	54.7	42	NA	NA
There is vaccination for hepatitis C	Hariho urukingo rw’indwara y’umwijima yo mu bwoko bwa C	Sultan 2018	19.3	7.8	43.5	NA
There is a treatment to cure hepatitis C.	Hariho umuti w’indwara y’umwijima yo mu bwoko bwa C.	NA	72	NA	NA	NA
